# Supplementary material for: Identification of photoperiod-induced specific miRNAs in the adrenal glands of Sunite sheep (Ovis aries)
Source: Front Vet Sci. 2022 Jul 22;9:888207. doi: 10.3389/fvets.2022.888207 (PMC9354845; doi:10.3389/fvets.2022.888207)
Supplement: Supplementary file 1 [file Data_Sheet_1.ZIP › Supplementary file/miRDeep2 file.zip!/miRDeep2 file/Know_Structure/LP42A2/oar-mir-29b-2.pdf]

miRBase precursor : oar-mir-29b-2
Total read count : 12799
oar-miR-29b read count : 11857
remaining reads : 942

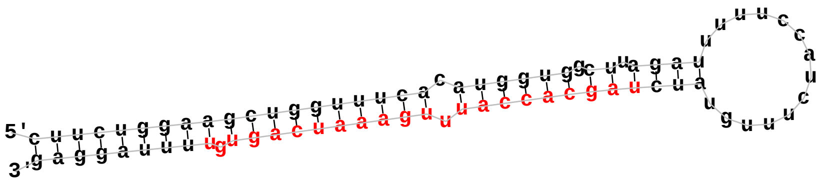

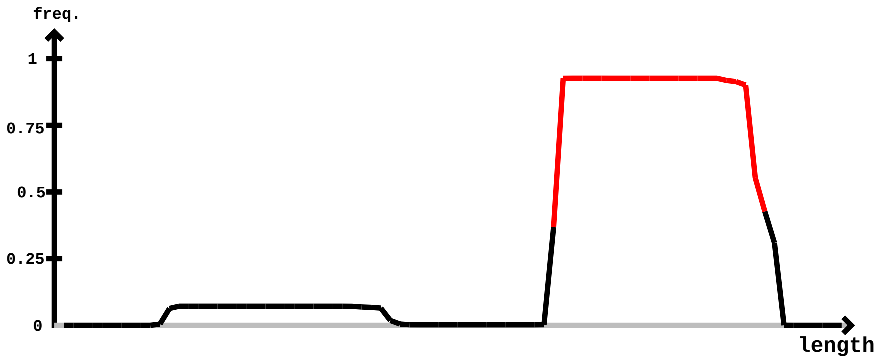

oar-miR-29b

```
5'- cuucuggaagcugguuucacaugguggcuuagauuuuuccaucuuuguaucuagcaccauuugaaaucaguguuuuaggag  -3'    exp
    (((((((((((((((((((.((((((.((.((((..............)))))))))))).)))))))))).)))))))))    reads   mm   sample
    .........gcugguuucacaugguggcu....................................................    4       0    seq
    .........gcugguuucacaugguggcuu...................................................    7       0    seq
    .........gcugguuucacaugguggcuua..................................................    4       0    seq
    .........gcugguuucacaugguggcuuag.................................................    5       0    seq
    .........gcugguuucacaugguggcuuaga................................................    36      0    seq
    .........gcugguuucacaugguggcuuagau...............................................    1       0    seq
    ..........cugguuucacaugguggcuu...................................................    24      0    seq
    ..........cugguuucacaugguggcuua..................................................    15      0    seq
    ..........cugguuucacaugguggcuuag.................................................    25      0    seq
    ..........cugguuucacaugguggcuuaga................................................    535     0    seq
    ..........cugguuucacaugguggcuuagau...............................................    133     0    seq
    ..........cugguuucacaugguggcuuagauu..............................................    19      0    seq
    ..........cugguuucacaugguggcuuagauuu.............................................    2       0    seq
    ...........ugguuucacaugguggcuu...................................................    2       0    seq
    ...........ugguuucacaugguggcuuag.................................................    3       0    seq
    ...........ugguuucacaugguggcuuaga................................................    48      0    seq
    ...........ugguuucacaugguggcuuagau...............................................    39      0    seq
    ...........ugguuucacaugguggcuuagauu..............................................    15      0    seq
    .................................uuuuuccaucuuuguau...............................    2       0    seq
    .................................uuuuuccaucuuuguauA..............................    1       1    seq
    .................................uuuuuccaucuuuguauU..............................    2       1    seq
    .................................uuuuuccaucuuuguauc..............................    18      0    seq
    .................................uuuuuccaucuuuguaucC.............................    1       1    seq
    ..................................uuuuccaucuuuguaucuagca.........................    1       0    seq
    .................................................ucuagcaccauuugaaauc.............    1       0    seq
    .................................................ucuagcaccauuugaaaucagu..........    5       0    seq
    ..................................................cuaCcaccauuugaaauc.............    1       1    seq
    ..................................................cuagcaccauuugaaauc.............    47      0    seq
    ..................................................cuagcaccauuugaaauca............    14      0    seq
    ..................................................cuagcaccauuugaaaucag...........    11      0    seq
    ..................................................cuagcaccauuugaaaucagu..........    2332    0    seq
    ..................................................cuagcaccauuugaaaucGgu..........    26      1    seq
    ..................................................cuagcaccauuugaaaucagug.........    1238    0    seq
    ..................................................cuagcaccauuugaaaucagugu........    1009    0    seq
    ..................................................cuagcaccauuugaaaucaguguu.......    5       0    seq
```

oar-miR-29b

```
cuucuggaagcugguuucacaugguggcuuagauuuuuccaucuuuguaucuagcaccauuugaaaucagugguuuaggag
...................................................uagcaccauuugaaauc.............    50     0     seq
...................................................uagcaccauuugaaauU.............    5      1     seq
...................................................uaUcaccauuugaaauc.............    1      1     seq
...................................................uagcaccauuugaaauca............    25     0     seq
...................................................uagcaccauuugaaaucG............    17     1     seq
...................................................uagcaccauuugaaaucag...........    29     0     seq
...................................................uagcaccauuugaaaucGg...........    126    1     seq
...................................................uagcaccauuugaaaucGgu..........    756    1     seq
...................................................uagcaccauuugaaaucagu..........    1323   0     seq
...................................................uagcaccauuugaaaucagug.........    387    0     seq
...................................................uagcaccauuugaaaucagugu........    488    0     seq
...................................................uagcaccauuugaaaucaguguu.......    3954   0     seq
...................................................uagcaccauuugaaaucaguguuu......    2      0     seq
....................................................agcaccauuugaaaucGgu..........    1      1     seq
....................................................agcaccauuugaaaucagu..........    1      0     seq
....................................................agcaccauuugaaaucagugu........    1      0     seq
....................................................agcaccauuugaaaucaguguu.......    2      0     seq
```
